# Rising Carbapenem MICs and Clinical Dose Optimization: Bridging Resistance Surveillance and Pharmacokinetic/Pharmacodynamic Strategies

**DOI:** 10.3390/antibiotics15090885

**Published:** 2026-09-09

**Authors:** Hülya Arık

**Affiliations:** Department of Infectious Diseases and Clinical Microbiology, Faculty of Medicine, Near East University, 99138 Nicosia, Cyprus; hulya.arik@neu.edu.tr

**Keywords:** carbapenem resistance, minimum inhibitory concentration, pharmacokinetics/pharmacodynamics, dose optimisation, therapeutic drug monitoring, extended infusion, antimicrobial stewardship, Eastern Mediterranean

## Abstract

The rise in carbapenem resistance is well documented, but a less conspicuous trend has received far less attention: the gradual upward shift in minimum inhibitory concentration (MIC) values among isolates that remain phenotypically “susceptible.” We propose the term carbapenem floor for this phenomenon: an intermediate zone in which standard dosing regimens become progressively less reliable at achieving target exposure, yet which remains invisible to breakpoint-based surveillance; we present this as a hypothesis-generating framework consistent with existing pharmacokinetic and epidemiological evidence rather than as an empirically established trend, since no systematic longitudinal quantification of carbapenem MIC drift has yet been undertaken. Because carbapenems exhibit time-dependent killing and their pharmacokinetic/pharmacodynamic (PK/PD) target has MIC as its denominator, even a shift within the susceptible range can substantially reduce target attainment; augmented renal clearance in critically ill patients deepens this erosion. This review integrates resistance surveillance evidence with PK/PD principles to evaluate prolonged infusion, loading doses, therapeutic drug monitoring (TDM), and empirical protocols derived from population pharmacokinetic models and local MIC distributions, examining for each the evidence and the practical constraints. We also consider the reverse relationship: failure to attain PK/PD targets appears to select for elevated MICs, suggesting that dosing practice may contribute to resistance selection and potentially influence resistance epidemiology, not only respond to it. Extending antibiogram reporting to include MIC distributions, and translating those data into dosing protocols through antimicrobial stewardship, is a feasible priority where TDM infrastructure is limited.

## 1. Introduction

Carbapenems remain among the last-line agents for serious infections caused by multidrug-resistant Gram-negative bacteria. Over the past two decades, however, the global rise in carbapenem resistance has begun to threaten the clinical reliability of the class. Data from the ATLAS global surveillance programme covering 2018–2022 show statistically significant increases in the detection of carbapenem-resistant Enterobacterales across the Asia-Pacific, European, Latin American and Middle East–Africa regions, with rates of difficult-to-treat resistance rising in all regions [1]. The geographical distribution of carbapenemase genes also varies: among meropenem-non-susceptible Enterobacterales, KPC-type enzymes were the most frequently identified carbapenemase in North America and Europe each year, whereas NDM-type enzymes became predominant in other regions by 2022 [1].

The Eastern Mediterranean, and Cyprus in particular, has not been spared. According to European Antimicrobial Resistance Surveillance Network (EARS-Net) data, the Republic of Cyprus was among the few European countries showing a statistically significant increasing trend in carbapenem resistance among invasive *Klebsiella pneumoniae* isolates between 2005 and 2010, reaching 16.4% by 2010 [2]; the surveillance position of Northern Cyprus differs and is considered in Section 5.3. Across the WHO Eastern Mediterranean Region more broadly, surveillance capacity remains limited; among bloodstream infections, the median proportion of carbapenem resistance was highest for *Acinetobacter* spp. (70.3%) and lowest for *Escherichia coli* (4.6%) [3].

A parallel trend attracts far less clinical attention but carries comparable importance: the gradual increase in MIC values even among isolates still classified as phenotypically susceptible. We propose to designate this phenomenon the carbapenem floor: an intermediate zone falling below the resistance breakpoint that nonetheless demands progressively higher antibiotic exposure. A related, though not directly confirmatory, observation comes from carbapenem-resistant *Pseudomonas aeruginosa* isolates from three centres in Türkiye, in which standard dosing regimens failed to reach PK/PD targets against other, non-carbapenem β-lactams (ceftazidime, cefepime, piperacillin/tazobactam) still reported as “susceptible” [4]. This illustrates the general phenomenon of susceptible-category exposure failure in a carbapenem-resistant background; it does not itself demonstrate carbapenem MIC drift, which, to our knowledge, has not been directly documented longitudinally for this class.

Carbapenems exert time-dependent antibacterial activity, and their clinical efficacy correlates directly with the fraction of the dosing interval during which free drug concentration remains above the MIC (%fT > MIC). While a baseline efficacy threshold of ≥40% fT > MIC is generally accepted, it is argued that in critically ill patients this target should be raised to 100% fT > MIC in order to improve survival and limit the emergence of resistance [5]. Physiological changes common in intensive care, augmented renal clearance, hypoalbuminaemia, and capillary leak can render dosing regimens predicted by standard population pharmacokinetic models inadequate [5].

The aim of this review is to synthesise epidemiological evidence regarding rising carbapenem MICs with time-dependent PK/PD principles, and to evaluate current strategies for clinical dose optimisation: extended and continuous infusion, therapeutic drug monitoring (TDM), and MIC-guided dosing. The intention is to set out an integrated framework linking resistance surveillance data to dosing decisions, drawing on both global and Eastern Mediterranean evidence.

### Scope and Literature Search

This is a narrative, not a systematic, review. PubMed/MEDLINE, Web of Science and Scopus were searched from database inception to 15 August 2026, combining the terms carbapenem, meropenem OR imipenem OR ertapenem OR doripenem, minimum inhibitory concentration OR MIC creep OR MIC shift, pharmacokinetic/pharmacodynamic OR PK/PD OR fT > MIC, dose optimisation OR extended infusion OR continuous infusion OR prolonged infusion, therapeutic drug monitoring OR TDM, Monte Carlo simulation OR probability of target attainment OR cumulative fraction of response, antimicrobial stewardship, and augmented renal clearance, using the Boolean structure (carbapenem terms) AND (MIC/resistance terms OR PK/PD terms OR dosing-strategy terms), with no starting-date restriction and English-language publication as the only formal filter. Randomised trials, systematic reviews and meta-analyses, surveillance programme reports and pharmacokinetic modelling studies were prioritised; reference lists of retrieved articles were hand-searched for additional sources, and author knowledge of the field supplemented the electronic search. Because this is a narrative synthesis rather than a systematic review, records were not logged against a PRISMA-type flow diagram at the time of the original search, and precise numbers of records retrieved and excluded at each stage cannot be retrospectively reconstructed; no formal risk-of-bias or certainty-of-evidence assessment (e.g., GRADE) was undertaken. What can be reported precisely is the outcome of the search process: 76 sources were ultimately cited, spanning 1998–2026, with the large majority published within the last ten years. To improve reproducibility despite this limitation, Table S2 (Supplementary Materials) provides a structured evidence table summarising study design, population, principal endpoint or finding, and key limitation for the randomised trials, meta-analyses and simulation studies that carry the review’s central clinical claims. Consequently, the hierarchy of evidence underlying different claims in this review is heterogeneous by design: guideline recommendations and large randomised trials are distinguished explicitly from single-arm simulations and observational cohorts throughout the text, and readers should weight claims accordingly rather than treating all citations as evidentially equivalent. This approach permits synthesis across heterogeneous evidence types but does not exclude selection bias, and the conclusions should be read accordingly. Because no systematic evaluation of within-susceptible-range carbapenem MIC shifts has, to our knowledge, been performed for this class in the way undertaken for vancomycin and *Staphylococcus aureus* (Section 2.2), the carbapenem floor concept introduced below is presented throughout as a hypothesis-generating conceptual framework consistent with existing pharmacokinetic and epidemiological evidence, and not as an established empirical finding; the title, figures and conclusions of this review are intended to be read with this qualification in mind.

## 2. Resistance Epidemiology and MIC Shift

### 2.1. Mechanisms of Carbapenem Resistance

Carbapenem resistance develops through several interacting mechanisms: (i) production of carbapenemase enzymes (such as KPC, NDM, VIM and OXA-48); (ii) loss or alteration of outer membrane porins, reducing permeability; (iii) overexpression of efflux pumps; and (iv) alteration of penicillin-binding proteins, although this last mechanism is infrequently observed in Enterobacterales [6,7]. In *Klebsiella pneumoniae*, deletion or mutation of the OmpK35/OmpK36 porins substantially impairs intracellular carbapenem penetration, while overexpression of the AcrAB-TolC efflux system lowers intracellular drug concentration and acts synergistically with enzymatic mechanisms [6].

The geographical distribution of carbapenemase genes differs by region: global surveillance data indicate that KPC-type enzymes predominate in North America and Europe, while NDM-type enzymes are more common elsewhere [1]. Within the Eastern Mediterranean Region, carbapenem resistance determinants show a heterogeneous distribution across countries [8].

Two features of this resistance mechanism profile are relevant here. A substantial proportion of carbapenem resistance is not carbapenemase-mediated: clinical and molecular epidemiology studies indicate that approximately half of CRE isolates do not carry a carbapenemase, developing resistance instead through combinations of ESBL or AmpC production with porin alterations [9]. In *P. aeruginosa*, the imbalance is more pronounced still, with non-enzymatic mechanisms, efflux and porin deficiency, together with AmpC overproduction, accounting for an estimated 85–95% of resistance [10].

These non-enzymatic mechanisms also produce graded rather than binary shifts in susceptibility. Whereas acquisition of a carbapenemase moves an isolate towards the resistant category in most instances, this is not universal: some carbapenemase-producing isolates, OXA-48-like producers in particular, can remain phenotypically categorised as susceptible or increased-exposure despite an impaired clinical response, which is precisely why current guidance has shifted back towards recommending routine carbapenemase testing (differentiating KPC, NDM, VIM, IMP and OXA-48-like enzymes) as a complement to, rather than a substitute for, phenotypic categorisation, so that mechanism-specific therapy can be selected where it is available (Section 2.1) [11,12]. Incremental porin loss, efflux upregulation and β-lactamase gene amplification, by contrast, raise the MIC by degrees, and can do so while the isolate remains formally susceptible. This provides a plausible biological substrate for the phenomenon described in Section 2.2, and suggests why carbapenemase-focused surveillance may under-detect it.

### 2.2. The “Carbapenem Floor”: MIC Shift Within the Susceptible Range

The gradual upward drift of MIC values among isolates that remain below the formal resistance breakpoint is of increasing clinical importance. Because such isolates are not classified as resistant, the drift generally remains invisible in surveillance reports, yet it may increase the risk of treatment failure in patients managed with standard dosing regimens. Among carbapenem-resistant *P. aeruginosa*, reduced potency has been observed even in isolates reported as susceptible to other, non-carbapenem β-lactams, with standard doses falling short of the expected probability of target attainment (PTA); this is illustrative of the broader exposure-failure phenomenon rather than direct evidence of carbapenem-specific MIC drift [4].

**Figure 1:**
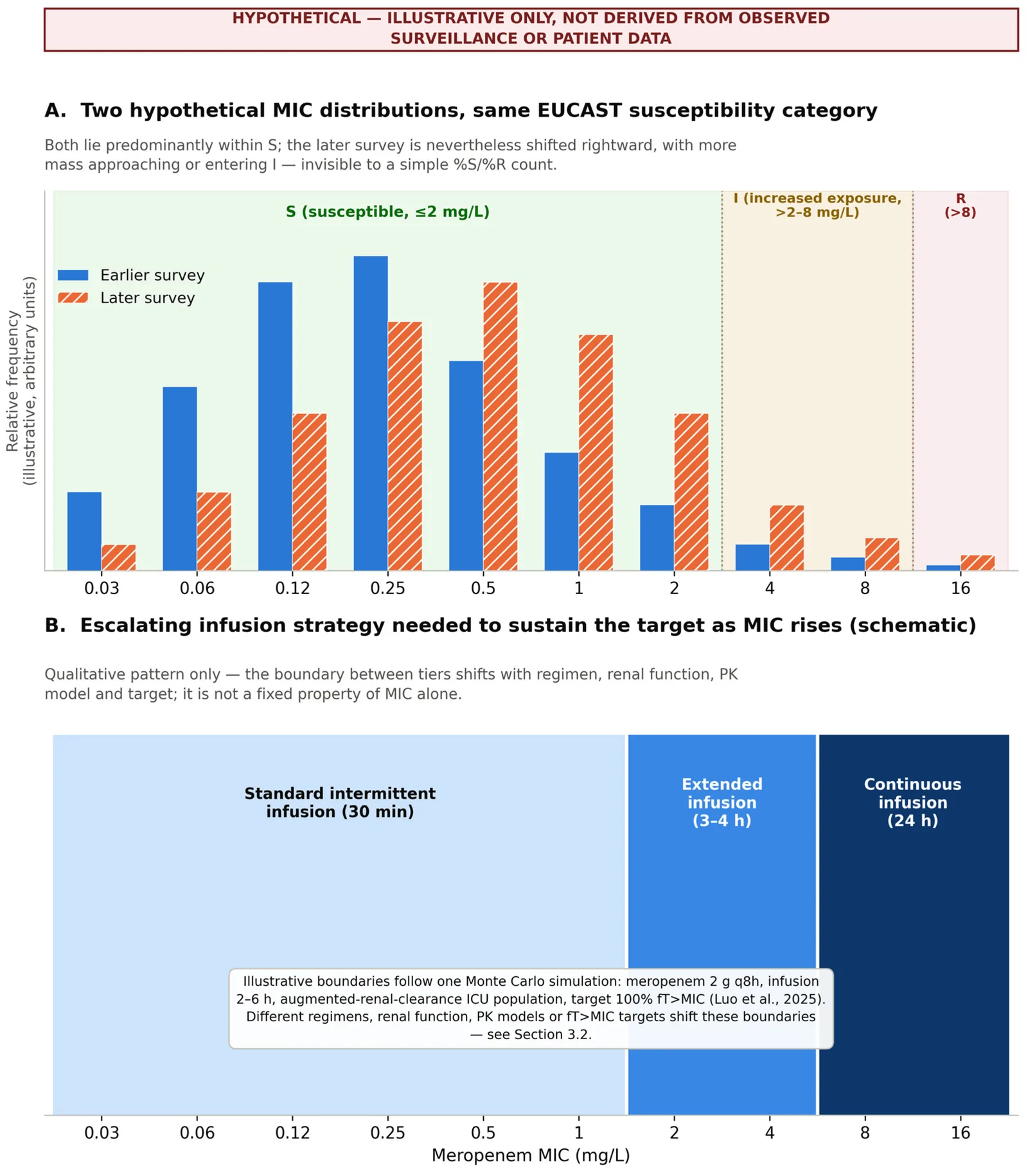
The carbapenem floor: what breakpoint-based reporting conceals (hypothetical illustration; not derived from observed surveillance data) [5].

We propose the term carbapenem floor for this phenomenon: an intermediate zone, created by upward shift in the MIC distribution within the susceptible category, in which standard dosing regimens become progressively less dependable. The concept rests on two observations: breakpoint-based surveillance renders shifts in the MIC distribution invisible, and, because the fT > MIC target depends directly on the MIC, an increase within the susceptible range makes target exposure appreciably harder to achieve (Section 3.2; Figure 1).

A parallel exists in the vancomycin literature, where MIC creep among *Staphylococcus aureus* has been debated for two decades. A systematic review and meta-analysis of 55 studies comprising 29,234 S. aureus isolates found mean vancomycin MICs of 1.23 mg/L (95% CI 1.13–1.33) by Etest and 1.20 mg/L (95% CI 1.13–1.28) by broth microdilution, with no evidence of a global MIC creep; both methods in fact showed a decrease over successive time strata [13]. A single-centre Portuguese study reached a complementary conclusion, arguing that MIC creep is a regional phenomenon that can only be assessed through evaluation of local susceptibility profiles, and that antibiograms based on actual MIC assays should be an essential element of local clinical management [14].

The vancomycin experience carries two lessons for the present argument. MIC drift should not be assumed universal, and its detection requires local, MIC-resolved surveillance rather than pooled international breakpoint data.

The two settings are not, however, mechanistically equivalent, and this bears on what should be expected. Reduced vancomycin susceptibility in *S. aureus* arises largely through cell-wall thickening, a costly adaptation that is unstable in the absence of drug pressure. Carbapenem MIC elevation in Gram-negative organisms arises instead through porin loss, efflux upregulation and β-lactamase gene amplification (Section 2.1), which are incremental and, in the case of gene amplification, readily reversible but also readily re-selected. A graded upward drift within the susceptible category is therefore more plausible for carbapenems than the vancomycin data would suggest by analogy alone.

The term is also worth distinguishing from related concepts already established in clinical microbiology, since these are sometimes used loosely and interchangeably. MIC creep denotes an observed, retrospectively documented upward shift in a population’s MIC distribution over time, as in the vancomycin literature discussed above; the carbapenem floor, by contrast, is a prospective hypothesis about where such creep, if it occurs, would matter clinically, namely within the susceptible category rather than at its boundary. The epidemiological cut-off value (ECOFF) separates wild-type isolates, lacking any acquired or mutational resistance mechanism, from non-wild-type isolates that carry one; ECOFFs are typically well below clinical breakpoints and are unaffected by dosing considerations, whereas the floor concerns isolates that are already non-wild-type (Section 2.1) and asks whether their clinical breakpoint category remains a reliable guide to achievable exposure. The floor is, finally, related to but not synonymous with the EUCAST “susceptible, increased exposure” category discussed in Section 2.4: that category is a fixed, dose-linked breakpoint zone, whereas the floor describes a dynamic tendency for isolates to accumulate within or below it over time. The concept is introduced here as a synthesising label for an implication of existing PK/PD and mechanistic evidence, not as a newly discovered or independently validated microbiological entity.

Table 1 summarises these distinctions for quick reference.

To our knowledge, no equivalent systematic evaluation has been undertaken for carbapenems and Gram-negative pathogens. The carbapenem floor is accordingly presented here not as an empirically validated finding but as a hypothesis implied by existing PK/PD and epidemiological data, requiring prospective testing through MIC-based surveillance.

Two hypothetical MIC distributions for an Enterobacterales population at successive time points, separated by the meropenem susceptibility breakpoint (≤2 mg/L): (A) Both distributions lie predominantly below the breakpoint, so the reported susceptibility rate is essentially unchanged, the quantity captured by conventional surveillance. The later distribution is nevertheless shifted rightward within the susceptible category. (B) The infusion duration required to achieve 100% fT > MIC at each MIC value: the same rightward shift moves a substantially larger proportion of isolates out of the range attainable by standard intermittent infusion and into the range requiring extended or continuous infusion. The contrast between the unchanged percentage in (A) and the changed dosing requirement in (B) is the phenomenon this review terms the carbapenem floor. Distributions in Panel A are entirely hypothetical and are not derived from any observed surveillance dataset. The infusion duration thresholds in Panel B are taken from a single Monte Carlo simulation of critically ill patients with augmented renal clearance, modelling meropenem 2 g every 8 h at varying infusion durations against the population pharmacokinetic model of Luo et al., a target of 100% fT > MIC, and creatinine clearance values of 140, 160, 180 and 200 mL/min; no loading dose was modelled, only the maintenance infusion strategies listed above [5]; they are shown to illustrate the qualitative principle that required infusion duration rises with MIC, not as universal infusion requirements applicable to other regimens, targets, renal function distributions or PK models, and should not be extrapolated to patients outside augmented renal clearance or to targets other than 100% fT > MIC without re-simulation.

### 2.3. Limitations of Current Surveillance Systems

Most existing resistance surveillance systems (EARS-Net, ATLAS, GLASS) rely on binary “susceptible/resistant” classification and generally do not report the continuous variability of MIC. This impedes early recognition of MIC shift, and leaves dosing decisions resting largely on static breakpoint tables. The use of a meropenem MIC threshold of ≥2 mg/L for carbapenemase gene screening within the ATLAS programme [1] demonstrates that MIC-based, drug-specific definitions are already employed in surveillance; nonetheless, distributional shifts below that threshold are not made visible in routine reporting.

A second, related limitation concerns detection methodology. Phenotypic carbapenemase assays such as the modified carbapenem inactivation method (mCIM) achieve high sensitivity in Enterobacterales but perform more variably in *P. aeruginosa*, in part because permeability barriers and efflux influence carbapenem diffusion during testing; isolates with porin loss may also mask inhibitor synergy and be misclassified [11]. Given that non-enzymatic mechanisms account for the majority of resistance in *P. aeruginosa* (Section 2.1), surveillance oriented towards carbapenemase detection is least sensitive in the setting where graded MIC shifts are most likely to arise.

### 2.4. EUCAST Breakpoints, “Susceptible, Increased Exposure”, and the Case for Breakpoint Revision

Under current EUCAST breakpoints, meropenem against Enterobacterales, *Pseudomonas aeruginosa* and *Acinetobacter* spp. is classified susceptible at MIC ≤ 2 mg/L, susceptible-increased-exposure at >2–8 mg/L, and resistant above 8 mg/L for non-meningitis indications; imipenem carries a narrower increased-exposure zone (>2–4 mg/L) against Enterobacterales other than Morganellaceae, while ertapenem has no increased-exposure category at all, being defined as susceptible at ≤0.5 mg/L and resistant above that value [15]. CLSI, by contrast, has set the meropenem and imipenem susceptible breakpoint for Enterobacterales at ≤1 mg/L (intermediate 2 mg/L, resistant ≥ 4 mg/L), roughly half the current EUCAST threshold [16,17]. An isolate reported susceptible under EUCAST criteria may therefore already fall in the intermediate range under CLSI, which is a further reason to state explicitly which breakpoint standard, version and organism-specific indication is assumed at each point in this review (Section 5.1).

The EUCAST “I” category was itself substantially redefined in 2019, from an ambiguous intermediate zone historically associated with uncertain therapeutic effect to an explicit statement that the isolate is expected to respond provided drug exposure at the infection site is increased, whether by a higher dose, more frequent administration or a prolonged infusion [18]. This is, in essence, the argument developed throughout the present review: that dose optimisation can recover clinical utility for isolates whose MIC lies above the standard-exposure threshold. Read this way, the meropenem increased-exposure range and the carbapenem floor described in Section 2.2 are not independent observations. The floor is, in substantial part, the trajectory of isolates drifting from standard-exposure susceptibility towards or into the increased-exposure category, a movement that breakpoint-anchored surveillance reports as unchanged susceptibility (Section 2.3) because S and I are both counted as “susceptible” in most routine reporting. CLSI’s intermediate category, lacking an equivalent structured dosing statement for most agents, does not support the same reading.

An alternative response to the same evidence is not to adjust dosing around a fixed breakpoint but to move the breakpoint itself. EUCAST announced in July 2025 that it would review breakpoints for carbapenems, alone and in combination with β-lactamase inhibitors, and subsequently issued a public consultation proposing to lower carbapenem breakpoints for Enterobacterales; a further round of consultation has since concluded, with a revised proposal intended for incorporation into a later version of the breakpoint tables [19]. Such revision is not a novel departure, including within the carbapenem class itself: CLSI’s own 2010 reduction in the meropenem and imipenem breakpoint, and its comparable reduction in the ertapenem breakpoint, were made for reasons closely paralleling those now cited by EUCAST, alongside historical reductions in fluoroquinolone, extended-spectrum cephalosporin and piperacillin-tazobactam breakpoints as accumulating clinical and PK/PD evidence outpaced the original criteria [17,20]. Lowering the meropenem breakpoint would reclassify some isolates currently read as susceptible or increased-exposure as resistant, achieving by redefinition of the standard what dose optimisation instead attempts by increasing achieved exposure against the existing one.

The two responses are better regarded as complementary than as competing solutions, and this review’s emphasis on dose optimisation should not be read as an argument against breakpoint revision. Revision protects the entire prescribing population from a threshold that no longer predicts outcome and requires no local infrastructure to act on, but it takes effect only after a formal, multi-year international process, offers no help to the critically ill patient whose isolate is already reported resistant, and does nothing for institutions slow to adopt a revised table. Dose optimisation can be deployed immediately at the bedside and, as Section 4.5 shows, can itself recover a substantial proportion of apparent resistance among non-carbapenemase-producing isolates; but it cannot correct a breakpoint that no longer reflects achievable exposure at any dose, and it places the burden of compensating for an outdated standard on individual clinicians rather than on the standard itself. In settings such as Northern Cyprus (Section 5.3), where adoption of a revised international breakpoint cannot be assumed to be prompt and institutional MIC data are the only available surveillance input, dose optimisation against the locally observed MIC distribution is likely to remain the more immediately actionable of the two approaches, without this implying that breakpoint revision is any less necessary once it becomes available.

### 2.5. The Inoculum Effect: A Second, Testing-Condition-Dependent Source of Elevated Effective MIC

Standard susceptibility testing is performed at a fixed inoculum, conventionally around 5 × 10^5^ CFU/mL, chosen for reproducibility rather than to represent bacterial density at the site of infection. β-lactams as a class can show attenuated activity, typically defined as a ≥four- to eight-fold rise in MIC, when tested against substantially higher inocula; carbapenems have historically been regarded as comparatively resistant to this effect relative to cephalosporins and penicillins, for which it is consistently demonstrable across multiple pathogens [21]. Recent mechanistic work indicates that this generalisation obscures heterogeneity directly relevant to the distinction drawn in Section 2.1.

In a study combining 106 clinical carbapenem-resistant Enterobacterales isolates with laboratory strains engineered to express individual carbapenemases (KPC, NDM, IMP, VIM, OXA-48 and SME), meropenem and ertapenem MICs rose linearly across the 10^5^–10^7^ CFU/mL range in every carbapenemase-producing isolate, with a median 10-fold increase and increases of up to 100-fold at the highest inoculum tested, while isolates whose resistance derived from porin loss alone showed essentially flat MICs across the same range [22]. At inocula still within the range considered acceptable for reproducible testing, 50% and 24% of carbapenemase-producing isolates changed susceptibility category for meropenem and ertapenem, respectively, and carbapenemase activity was detectable in the culture supernatant, consistent with an enzymatic rather than a permeability-based mechanism [22]. This sharpens rather than contradicts the distinction drawn in Section 2.1: the graded, incremental drift proposed here as the carbapenem floor arises from porin loss, efflux upregulation and non-carbapenemase β-lactamase amplification, mechanisms shown in the same study to be essentially inoculum-independent, whereas the inoculum effect is disproportionately a property of carbapenemase producers. The two phenomena identify different, non-overlapping subpopulations in which a standard-inoculum MIC misrepresents the exposure actually required, and neither can be inferred from the other.

A second line of evidence extends the effect beyond carbapenemase producers specifically. Using larger test volumes and higher inocula, meropenem MICs against *Klebsiella pneumoniae* rose two- to 16-fold and two- to eight-fold, respectively, sufficient to reclassify several isolates from susceptible to resistant; hollow-fibre infection models simulating standard clinical dosing at 10^6^ versus 10^8^ CFU/mL starting inoculum showed regimens adequate at the lower, standard-testing-like density failing to suppress growth at the higher one [23]. Because deep-seated infections such as abscesses, empyema and endocarditis-like vegetations routinely present bacterial densities of 10^8^–10^9^ CFU/mL or more, several orders of magnitude above the standard testing inoculum, the reported MIC in such infections may understate the exposure actually required. This has been demonstrated directly in a simulated high-density endocardial vegetation model of Enterobacter cloacae complex infection at 10^9^ CFU/g: cefepime failed against every isolate tested, with treatment-emergent MICs exceeding 64 mg/L through inducible β-lactamase expression, while meropenem succeeded with extended infusion against susceptible strains but failed against carbapenemase producers despite nominally adequate exposure; only meropenem-vaborbactam retained consistent bactericidal activity across isolates [24].

The clinical implication runs parallel to, but is mechanistically distinct from, both the carbapenem floor (Section 2.2) and the increased-exposure category discussed above (Section 2.4). All three describe ways in which a single MIC value obtained under standardised laboratory conditions can understate the exposure required for cure, but they operate through different routes: the floor is a population-level drift detectable at standard inoculum; the increased-exposure category is a dosing-linked breakpoint zone; and the inoculum effect is a within-isolate discrepancy between the reported MIC and the MIC operative at a specific, high-density infection site. Their combination is of particular concern in carbapenemase-producing organisms causing deep-seated infection, where a reported susceptible or increased-exposure MIC may substantially overstate achievable efficacy regardless of the dose optimisation strategies discussed in Section 4, and where source control, rather than dose escalation alone, is likely to remain the dominant determinant of outcome.

### 2.6. MIC Measurement Uncertainty and Requirements for Longitudinal Surveillance

The framework proposed in Section 2.2, Section 2.4 and Section 2.5 depends on detecting comparatively small, sustained shifts in MIC distributions over time. This must be reconciled with the inherent technical variability of MIC testing itself. Routine quality control ranges for broth microdilution accept a spread of one doubling dilution around the target value as normal assay variability; a single measured MIC can therefore differ from the ‘true’ underlying value by a factor of two on technical grounds alone, before any biological change has occurred. An apparent one-dilution rightward shift in a surveillance dataset, which is the order of magnitude the carbapenem floor concept is concerned with, is accordingly indistinguishable from assay noise unless the surveillance design used to detect it can rule this out.

Several further sources of variability compound this. Broth microdilution, gradient diffusion (Etest) and automated platforms (e.g., Vitek, Phoenix, BD systems) do not always agree within one dilution for carbapenems, particularly near the breakpoint; reagent lot, media batch, inoculum preparation and incubation conditions each contribute additional variance; and isolates tested only across a limited dilution range generate censored values (reported as, for example, ‘≤0.25’ or ‘≥32 mg/L’) that are handled inconsistently when pooled into a distribution unless a pre-specified convention is applied. A change in testing platform or media supplier at a single institution partway through a surveillance period can by itself generate an apparent ‘MIC creep’ that has no biological correlate, a possibility that cannot be excluded post hoc and must instead be designed against prospectively.

A surveillance protocol capable of testing the carbapenem floor hypothesis should therefore specify, and hold constant or explicitly document changes in, the following: the reference method used (preferably broth microdilution performed and quality-controlled to a single standard, CLSI or EUCAST, rather than a mixture of methods); routine external quality control against reference strains; a pre-specified rule for handling censored MIC values in distributional analysis; a first-isolate-per-patient-per-surveillance-period rule and exclusion of duplicate isolates, to prevent serial isolates from a single infection from distorting the population distribution; stratification by specimen source, ward or unit and patient population, since case mix drift can itself shift an aggregate MIC distribution independent of any change in the organisms’ underlying susceptibility; a documented record of any change in testing platform, media lot or laboratory methodology across the surveillance period; and carbapenemase or resistance-mechanism characterisation of a defined subsample, so that an observed shift can be attributed to genuine non-enzymatic drift of the kind the floor concept describes (Section 2.1) rather than to a change in the underlying prevalence of carbapenemase-producing isolates (Section 2.5). The minimum isolate numbers conventionally recommended for reporting a stable cumulative susceptibility percentage, at least 30 isolates per organism-drug combination per analysis period, still carry appreciable sampling error for combinations near 50% susceptibility, and smaller institutional collections are likely to require aggregation across multiple years before a genuine distributional shift can be distinguished from year-to-year sampling variability [25,26].

None of this invalidates the framework proposed here; it specifies what would be required to test it properly, which is itself a gap identified in Section 6. In its absence, the case for the carbapenem floor rests, as stated throughout, on biological plausibility and PK/PD simulation rather than on a demonstrated, technically robust longitudinal shift, and it should continue to be read that way until such surveillance exists.

## 3. Pharmacokinetic/Pharmacodynamic Foundations

### 3.1. Time-Dependent Killing and the fT > MIC Target

Like all β-lactams, carbapenems exhibit time-dependent bactericidal activity: their killing efficacy is determined not by peak concentration but by the duration for which free (unbound) drug concentration exceeds the MIC. The fundamental PK/PD index describing this relationship is the percentage of the dosing interval during which free concentration remains above the MIC (%fT > MIC) [27].

The required fT > MIC differs across β-lactam classes. In non-neutropenic patients, targets of 50–60% for penicillins, 60–70% for cephalosporins, and 40–50% for carbapenems have been proposed [27]. The comparatively shorter threshold for carbapenems is attributed to this being the only β-lactam subclass that exhibits a post-antibiotic effect (PAE) against Gram-negative organisms [28].

These classical thresholds were derived chiefly from bacteriostatic and near-maximal-bactericidal endpoints in non-neutropenic animal infection models, and at least three conceptually distinct targets should be kept separate rather than treated as interchangeable: a bacteriostatic or near-maximal-kill target (the 40–70% fT > MIC range in Table 2), a more aggressive clinical-efficacy target advocated for selected critically ill populations (100% fT > MIC), and a still more aggressive resistance-suppression target derived from observational data (100% fT > 4× MIC, Section 4.6). These are not simply points on a single severity scale: they rest on different classes of evidence (animal PK/PD models, single-population simulation, and observational cohort studies, respectively), and the optimal target for a given patient plausibly depends on the drug, the infecting pathogen, the site of infection, immune status, illness severity and toxicity risk, none of which is captured by a single universal number. Consistent with this, a 100% fT > MIC target has been argued for specific populations, such as critically ill patients with augmented renal clearance modelled by Luo et al. [5], rather than established as a target that applies uniformly to all critically ill patients; the strength of this recommendation should not be overstated beyond the populations in which it has actually been evaluated.

### 3.2. The Determinant Role of MIC in Dose Adequacy

Because the MIC forms the denominator of the fT > MIC target, the drug exposure (or, at fixed dose, the infusion duration) required to sustain a given fT > MIC target rises with MIC in a manner that is not linear in dose, so that equal absolute increases in MIC do not require equal absolute increases in exposure to compensate. The carbapenem floor described in Section 2.2 is therefore a direct mechanism by which standard dosing regimens lose their probability of target attainment.

This relationship has been illustrated concretely in one Monte Carlo simulation, whose specific parameters (dose, infusion duration, PK model, creatinine clearance range and 100% fT > MIC target) are stated in the Figure 1 legend and should be regarded as an illustrative case rather than a generalisable rule: in critically ill patients with augmented renal clearance, a meropenem regimen of 2 g every 8 h administered over 2–3 h achieved ≥90% PTA (100% fT > MIC) for MIC ≤ 2 mg/L, whereas intensified regimens (2 g every 8 h over 4–6 h, or continuous infusion) became necessary to reach targets across the MIC range of 4–8 mg/L [5]. Regimens, targets and PK models differ across institutions and patient populations, and the general principle, that required exposure rises with MIC, should be distinguished from the specific numerical thresholds of this one simulation, which do not transfer automatically to other dosing regimens, renal function distributions or fT > MIC targets.

### 3.3. Altered Pharmacokinetics in the Critically Ill

Intensive care patients exhibit a range of pathophysiological changes that render carbapenem pharmacokinetics unpredictable:

- Augmented renal clearance (ARC): Defined as creatinine clearance >130 mL/min/1.73 m^2^, ARC is far from exceptional. A systematic review and meta-analysis of 70 studies found a pooled prevalence of 39% (95% CI 34.9–43.3) among critically ill patients, rising to 74% (95% CI 55–87) in neurocritical care and 58% (48–67) in trauma ICUs, with 36% and 33% in mixed and sepsis ICUs respectively [29]. Risk factors identified on pooled analysis were younger age (OR 0.95 per year; 0.93–0.96), male sex (OR 2.36; 1.28–4.36) and trauma (OR 2.60; 1.21–5.58) [30]. ARC is consistently associated with subtherapeutic antibiotic plasma concentrations [31], and, critically for detection, occurs despite a normal serum creatinine concentration [32].
- Increased volume of distribution: Capillary leak and aggressive fluid resuscitation in sepsis expand the volume of distribution of hydrophilic drugs, lowering plasma concentrations.
- Hypoalbuminaemia: For carbapenems with higher protein binding (e.g., ertapenem), this increases the free fraction and accelerates clearance.
- Organ dysfunction and renal replacement therapy: Conversely, in renal failure or during continuous renal replacement therapy (CRRT), drug accumulation and toxicity become the concern.

Higher PK/PD targets are not obtained without cost. Excessive β-lactam exposure, particularly in the presence of renal dysfunction, has been associated with neurotoxicity: a systematic review of cefepime-induced neurotoxicity found renal impairment in 80% of affected patients and dosing that exceeded recommended levels in nearly half, though a substantial minority developed neurotoxicity despite nominally appropriate dosing, indicating that standard doses do not guarantee safety in all patients [33]. Although the evidence base for carbapenem-specific neurotoxicity at high exposure is less extensive than for cefepime, the general principle, that renal function should bound how aggressively a PK/PD target is pursued rather than being treated only as a determinant of clearance to be overcome by dose escalation, applies to the class as a whole and is a necessary counterweight to the dose-optimisation emphasis of this review. No single standard dose can therefore be appropriate for all critically ill patients: dosing must be individualised to the patient’s current physiology, renal function and toxicity risk, and the pathogen’s MIC, which is the rationale for the strategies considered in Section 4.

A less obvious point concerns which patients are affected. Those at greatest risk of underexposure are not necessarily the ones identified as high risk on clinical grounds. ARC is associated with younger age, fewer comorbidities and lower illness severity scores [32], and it is invisible on the serum creatinine that clinicians routinely use to judge renal handling—a pattern established in a multicentre observational study of renal function in critically ill patients with normal plasma creatinine concentrations [34]. The patient who appears physiologically well, with unremarkable laboratory values, may be exactly the one clearing carbapenem too fast to maintain target exposure. Systematic screening, whether by measured creatinine clearance or by validated tools such as the ARC or ARCTIC scores, is therefore recommended in high-risk ICU patients rather than relying on clinical impression [32]. Dosing regimens specifically recommended for patients with ARC have been the subject of a dedicated systematic review [35].

## 4. Dose Optimisation Strategies

The two problems set out in Section 3, rising MIC values and unpredictable pharmacokinetics in the critically ill, increasingly render standard intermittent infusion regimens inadequate. This section examines the principal strategies used to achieve target exposure.

### 4.1. Extended and Continuous Infusion

Given time-dependent killing kinetics, distributing the same daily dose over a longer period is the simplest and least costly intervention that directly increases fT > MIC. Replacing standard 30 min infusion with 3–4 h extended infusion, or with 24 h continuous infusion, markedly improves PTA, particularly against high-MIC isolates.

The status of this strategy in guidance has recently changed, within a specific scope that should be stated precisely. The 2026 Surviving Sepsis Campaign guidelines upgraded prolonged infusion of β-lactams for maintenance dosing, following an initial loading dose, from a conditional suggestion to a strong recommendation over bolus administration, on moderate-certainty evidence, for adults with sepsis or septic shock [36]; the recommendation does not extend, as stated, to all carbapenem-treated patients or infections outside that population. Within this scope, prolonged infusion is a recommended default for sepsis, which shifts the relevant question from whether to adopt it to how to implement it reliably; this default-adoption argument and the targeted-subgroup argument developed later in this section (Section 4.1.1) are not in tension, since a guideline-level default for an unselected septic population is compatible with the pharmacological benefit concentrating disproportionately in the subset with high-MIC pathogens or augmented renal clearance, exactly as an averaged clinical-trial effect diluted across an unselected population would predict. Extended (3–4 h) and continuous (24 h) infusion are, in addition, evaluated together in much of the literature discussed below but differ materially in their evidence base, feasibility and chemical-stability constraints (Section 4.1, later paragraphs); where the distinction matters for a specific claim, it is stated explicitly.

#### 4.1.1. Clinical Trial Evidence

Two adequately powered randomised trials have now tested whether this pharmacological advantage translates into clinical benefit, and their results warrant careful reading.

The MERCY trial randomised 607 critically ill patients with sepsis across 31 ICUs in four countries to continuous versus intermittent meropenem, with a composite primary outcome of mortality and emergence of pandrug-resistant or extensively drug-resistant bacteria. It found no benefit from continuous administration [37].

These trials followed two earlier studies from the same group: BLING I, a double-blind randomised trial in severe sepsis [38], and BLING II, a larger multicentre randomised trial [39], neither of which was powered to detect a mortality difference. The BLING III trial subsequently randomised 7202 critically ill adults with sepsis across seven countries to continuous versus intermittent infusion of meropenem or piperacillin/tazobactam at equivalent daily doses, with 90-day all-cause mortality as the primary outcome. Mortality was 24.9% with continuous versus 26.8% with intermittent infusion (OR 0.91; 95% CI 0.81–1.01; *p* = 0.08), numerically favourable but not statistically significant on the primary analysis, although a prespecified adjusted analysis reached *p* = 0.04 [40]. Clinical cure was improved, and no clear increase in resistant-organism acquisition was observed. The accompanying meta-analysis estimated a number needed to treat of 25 to save one additional life at 90 days, with a 99.1% posterior probability of lower 90-day mortality [40].

A separately reported UK cohort of BLING III (*n* = 2900) showed baseline characteristics and outcomes closely matching the global trial, with 90-day mortality of 26.7% versus 29.3% (OR 0.88; 95% CI 0.75–1.04); all outcomes again favoured continuous infusion on point estimate without reaching significance [41].

The discordance between these trials is not irreconcilable. MERCY was substantially smaller and used a composite endpoint incorporating resistance emergence; BLING III was more than ten times larger and, while missing statistical significance on its primary endpoint, showed all outcomes with point estimates favouring continuous infusion. A subsequent systematic review and meta-analysis with trial sequential analysis has attempted to reconcile these findings [42]. The reasonable current position is that continuous infusion is unlikely to be harmful, probably confers a modest benefit, and that the magnitude of any benefit is small enough to have eluded even a 7000-patient trial.

This bears on the present argument. A modest average effect across an unselected ICU population is what would be expected if the intervention matters chiefly in the subset of patients where target attainment is at risk: those infected by high-MIC organisms, or with augmented renal clearance. Trials randomising irrespective of pathogen MIC dilute the signal that MIC-guided selection would concentrate. The implication is not that prolonged infusion is ineffective, but that it should be targeted.

#### 4.1.2. Chemical Stability and Practical Constraints

Chemical stability is, however, a critical constraint on this strategy. Carbapenems are the least stable of the β-lactams: in concentrated aqueous solution they undergo >10% degradation within ≤5–6 h at 25 °C, whereas ceftazidime, piperacillin and temocillin remain stable for 24 h or longer [43]. For this reason, carbapenems have in practice largely been restricted to 3 h extended infusion [43].

The stability window varies markedly with concentration, temperature and generic product, which is the principal source of inconsistency in the literature. Franceschi and colleagues found a 5 mg/mL generic meropenem solution stable for up to 8 h between 25 and 35 °C, falling to 5 h at 40 °C, and concluded that continuous infusion is applicable where ambient temperature remains below 35 °C provided the solution is renewed every 6–8 h [44]. Katip and colleagues evaluated two further generic brands at higher concentrations and reported shorter windows: for brand A, a 10 mg/mL solution was stable for up to 10 h at 25 °C but only 5 h at 30 °C and 4.5 h at 35 °C, while its 20 mg/mL solution was stable for 6 h at 25 °C, 5 h at 30 °C and 3 h at 35 °C; brand B at 10 mg/mL was stable for 9 h at 25 °C, 5 h at 30 °C and 3 h at 35 °C [45]; the authors note that ambient temperatures of 35 °C are common on medical wards in tropical countries for much of the year, so this shortened stability window is of direct practical relevance rather than a laboratory extreme. The dependence is even more pronounced for imipenem: 5 mg/mL solutions remained stable for 6 h at 25, 30 and 40 °C, whereas 10 mg/mL solutions lost stability within less than 1 h at 30 and 40 °C [46]. These findings are summarised in Table 3.

Comparable temperature dependence has been reported for doripenem, imipenem and meropenem at elevated room temperatures [48], and differences between generic products extend beyond stability to in vitro potency and dissolution characteristics [49,50]. A protocol validated for one product cannot be assumed to transfer to another.

For practice, continuous infusion is feasible for meropenem but requires cooling or frequent bag replacement: in cooled elastomeric devices, a 1% meropenem solution showed a mean 24 h recovery of 90.3%, and patients received more than 95% of the maximum deliverable dose [47]. More consequentially, stability data are not directly transferable between institutions: because the generic product used, the concentration prepared and the ambient ward temperature all alter the result, each institution must validate under its own conditions. This constraint is also a major reason for the heterogeneity of “continuous infusion” studies in the literature, in which infusion durations range from 30 min to true continuous administration.

The point carries particular weight in warm climates. Ward temperatures of 30–35 °C during Mediterranean summers fall within the range where the stability of both meropenem and imipenem deteriorates sharply, a consideration rarely addressed in guidance derived from temperate-climate institutions.

### 4.2. Loading Dose Strategy

In patients receiving continuous infusion, time to steady state is prolonged, creating a risk of subtherapeutic exposure during the first hours of therapy. A loading dose administered before commencing continuous infusion is therefore recommended. The loading dose additionally compensates for the expanded volume of distribution in sepsis and can be given independently of renal function [51].

### 4.3. Therapeutic Drug Monitoring (TDM)

β-lactam TDM offers the possibility of individualising dose by directly measuring pharmacokinetic variability in the critically ill patient. The systematic review and meta-analysis by Pai Mangalore and colleagues (11 studies, *n* = 1463) found that TDM-guided dosing, compared with standard dosing, significantly improved clinical cure (RR 1.17; 95% CI 1.04–1.31), microbiological cure (RR 1.14; 95% CI 1.03–1.27) and PK/PD target attainment (RR 1.85; 95% CI 1.08–3.16), while reducing treatment failure (RR 0.79; 95% CI 0.66–0.94) [52].

The limitations of this evidence base are substantial. The same meta-analysis demonstrated no significant effect of TDM on all-cause mortality or on ICU/hospital length of stay [52]. The randomised DOLPHIN trial, which compared model-informed precision dosing (MIPD) of β-lactams and ciprofloxacin against standard dosing in 388 ICU patients, likewise found no significant difference in clinical outcomes, with ICU length of stay as its primary endpoint [53]. Zeggil and Dalton subsequently incorporated the β-lactam data from DOLPHIN into the Pai Mangalore meta-analysis and re-analysed it, reporting that the effect estimate for mortality moved still closer to the null, concluding that evidence on hard clinical endpoints remains insufficient to support widespread adoption of TDM [54].

A subsequent, exploratory subgroup analysis of DOLPHIN examined which patients might benefit, and found lower 28-day mortality with MIPD among patients with a baseline SOFA score below 8 (OR 0.40; 95% CI 0.17–0.88) [55]. This finding should not, on its own, be used to define a preferred TDM population: it was not a prespecified primary comparison, and the same subgroup analysis has been reported to be accompanied by a longer ICU stay in this lower-SOFA group and by a less favourable, statistically non-significant trend among patients with higher SOFA scores, a trade-off that a mortality-only summary obscures [55]. As a hypothesis-generating subgroup finding it requires prospective confirmation in a trial designed to test it, and it should be read as suggestive that TDM benefit may not simply track illness severity, rather than as evidence that TDM should be targeted to less severely ill patients in practice.

Current guidance reflects this uncertainty. In contrast to the strong recommendation for prolonged infusion, the 2026 Surviving Sepsis Campaign guidelines only suggest antimicrobial TDM on a case-by-case basis in selected patients, on very-low-certainty evidence, and specify that selection should rest on clinical features, drug class, TDM availability and local pathogen and resistance patterns [36]. The inclusion of local resistance patterns among the stated selection criteria is notable: guidance now explicitly conditions an individual dosing decision on institutional surveillance data, which is the linkage this review examines in Section 5.

TDM is therefore better positioned as a tool directed towards defined subgroups than as a routine standard: patients with ARC, those receiving CRRT, cases infected with high-MIC pathogens, and patients failing to respond to therapy. The DOLPHIN subgroup finding above is not included in this list for the reason just given: it is hypothesis-generating rather than a basis for selecting patients by illness severity.

A separate randomised trial of TDM-based dose optimisation of piperacillin/tazobactam likewise failed to demonstrate improvement in sepsis-related organ dysfunction [56], further underlining that target attainment and clinical benefit have not proved straightforwardly equivalent. The DOLPHIN protocol had prespecified ICU length of stay rather than mortality as its primary endpoint [57], a choice that limits what the trial can say about survival.

The timing of TDM is also attracting increasing attention. In a prospective study of 297 infection episodes, delayed TDM was associated with reduced odds of clinical cure, while patients in whom dose, frequency or infusion duration were increased showed lower 30-day mortality [58]. Several practical dimensions of TDM implementation, beyond the question of which patients to target, materially affect whether it can deliver the benefits described above: whether total or unbound (free) drug concentration is measured, since only the unbound fraction is pharmacologically active and protein binding varies with hypoalbuminaemia (Section 3.3); assay turnaround time, which determines whether a result can still change the next dose or only retrospectively explains treatment failure; the sampling strategy used to estimate fT > MIC from a limited number of levels; the use of Bayesian forecasting to combine a population PK model with sparse patient-specific levels rather than relying on a single trough or peak; the need for repeat sampling when renal function is changing rapidly, as is common in the critically ill (Section 3.3); pre-defined toxicity thresholds alongside efficacy targets (Section 3.1); and whether the MIC used to interpret a given level is a directly measured value for the infecting isolate or an assumed epidemiological value, which affects the confidence that can be placed in a single TDM-guided dose adjustment [51]. Table 4 summarises which patient groups are best placed to benefit from TDM on current evidence; because several of these judgements rest on exploratory or indirect evidence, this should be read as an evidence-informed prioritisation rather than a validated algorithm.

### 4.4. MIC-Guided Dosing and Monte Carlo Simulation

In settings where individual TDM is not accessible, population pharmacokinetic (popPK) models and Monte Carlo simulation offer a practical alternative for constructing empirical dosing protocols based on local MIC distribution. In this approach, the MIC distribution of an institution’s own isolates is entered as simulation input, PTA values for different dosing regimens are calculated, and the regimen achieving the ≥90% threshold is adopted as the empirical standard. Simulation studies conducted against carbapenem-resistant *P. aeruginosa* isolates from Türkiye demonstrate the effectiveness of this method in generating dosing recommendations adapted to a regional resistance profile [4].

Implementation turns on two distinctions. The first concerns the choice of index. PTA describes the probability of reaching a target against a specified MIC, whereas the cumulative fraction of response (CFR) integrates PTA across the entire MIC distribution of a pathogen population, and is therefore the appropriate metric when the pathogen MIC is not yet known and the question is empirical coverage. CFR is computed directly from the local MIC distribution, which makes the quality of institutional surveillance data the limiting input rather than the model itself. Where a modest upward shift in the MIC distribution occurs, PTA at any single MIC is unchanged while CFR falls, so CFR is also the index in which the carbapenem floor would first become visible.

The second concerns model selection. Population PK models are derived in defined populations, and their covariate structures reflect the case mix in which they were built; a model developed in a general ICU cohort may misestimate clearance in a population with a high prevalence of augmented renal clearance (Section 3.3). Where a model incorporating measured creatinine clearance is available, it is preferable to one relying on estimated glomerular filtration rate, since ARC is precisely the condition under which serum creatinine misrepresents renal handling [34]. Selecting a model whose derivation population resembles the institution’s own, and stating that choice explicitly in the protocol, is a minimum requirement for the approach to be defensible.

The workflow just described presupposes several methodological choices that determine whether its output is defensible, and these should be made explicit rather than left implicit. A minimum number of isolates per organism-specimen combination should be pre-specified before a distribution is used for simulation, recognising that even the conventionally cited minimum of 30 isolates carries appreciable sampling error and that smaller institutional volumes are likely to require multi-year aggregation (Section 2.6) [25,26]; a first-isolate-per-patient rule and exclusion of duplicate isolates should be applied for the same reasons set out in Section 2.6; the analysis period and its planned reassessment interval should be fixed in advance; results should be stratified by infection source and, where numbers allow, by ward or unit, since the case mix can shift an aggregate distribution independently of any change in organism susceptibility; the population PK model’s derivation cohort should be validated against the local population before adoption, not merely selected for covariate convenience (as already noted above); and the protocol should specify who is responsible for periodic reassessment and for approving changes to the institutional dosing table, since this is a clinical governance function and not only a technical one. None of these requirements is met simply by having access to MIC data and simulation software, and the ‘low cost’ characterisation of this approach elsewhere in this review (Section 5.2 and Section 7) refers specifically to the absence of new drug acquisition or TDM assay infrastructure; reliable MIC testing, data management, simulation expertise, dedicated pharmacy time and local stability validation (Section 4.1) are themselves real resource requirements that should not be understated.

**Figure 2:**
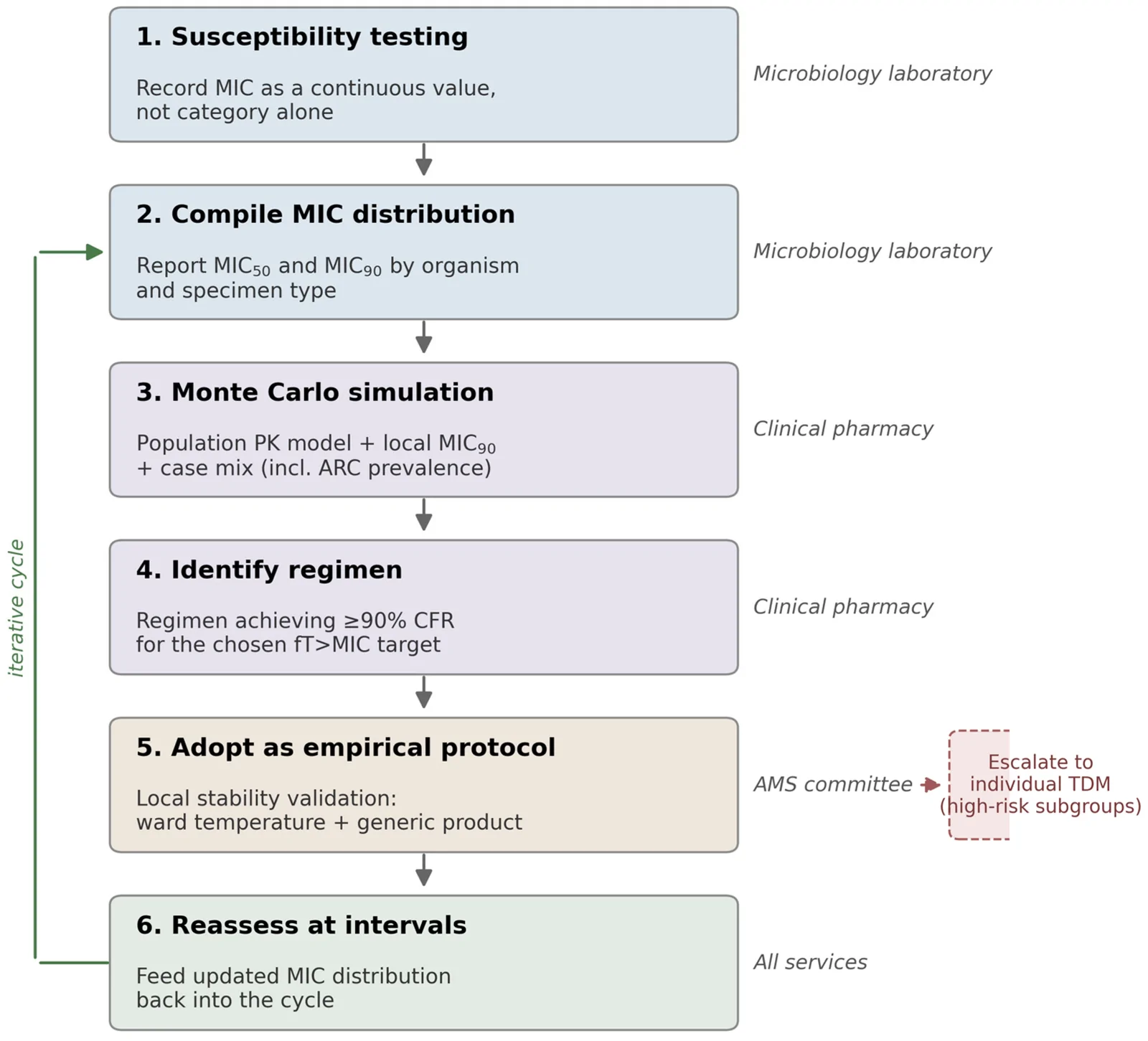
Linking local surveillance to an institutional dosing protocol. Box colour indicates the responsible service: blue = microbiology laboratory; lavender = clinical pharmacy; tan = antimicrobial stewardship (AMS) committee; green = all services; the dashed red box denotes escalation to individual therapeutic drug monitoring (TDM) for high-risk subgroups.

The output of this exercise is a dosing table rather than a single regimen: for each organism and specimen type, the regimen achieving CFR ≥90% against the current local MIC distribution. Because that distribution shifts, the table has a shelf life, and periodic recomputation is part of the method rather than an optional refinement (Figure 2).

This approach converts surveillance data directly into a dosing decision, and represents the most concrete link between resistance monitoring and dose optimisation available at present.

A six-stage workflow with the responsible service indicated for each stage. Susceptibility testing records MIC as a continuous value rather than category alone (1), permitting periodic compilation of the institutional MIC distribution (2). Monte Carlo simulation combines a population PK model with the full local MIC distribution, not the MIC90 alone, and the institution’s case mix, including local ARC prevalence (3), to identify the regimen achieving CFR ≥ 90% for the chosen fT > MIC target (4); MIC90 is reported alongside the distribution as a summary statistic for antibiogram readers (Section 5.1) but is not itself a sufficient simulation input, since CFR is by definition an integration of PTA across the entire distribution rather than a calculation at a single MIC value (Section 4.4). Adoption as institutional empirical protocol requires local stability validation, since ward ambient temperature and the generic product in use both alter the feasible infusion duration (5). Periodic reassessment feeds an updated MIC distribution back into stage 2, making the process iterative rather than a single exercise (6). A side branch indicates escalation to individual TDM for the high-risk subgroups defined in Section 4.3.

### 4.5. Combination Therapy and Alternative Agents

Where the MIC has risen beyond what dose escalation can overcome (true carbapenem resistance), dose optimisation alone is insufficient. In such scenarios, newer β-lactam/β-lactamase inhibitor combinations (ceftazidime-avibactam, meropenem-vaborbactam, imipenem-relebactam) or colistin-based combinations come into consideration. The simulation data cited above are relevant here: in patients with augmented renal clearance, no meropenem regimen achieved adequate cumulative fraction of response (CFR ≥ 90%) against *Acinetobacter baumannii* or *P. aeruginosa*, indicating the need for alternative or combination therapy for these pathogens [5].

The activity of these agents is, however, mechanism-dependent in ways that reinforce the argument of Section 2.1. In US surveillance covering 2018–2022, ceftazidime-avibactam was the most active agent against CRE isolates (84.2% susceptible), followed by meropenem-vaborbactam (81.9%) and imipenem-relebactam (74.8%); all three were highly active against KPC producers, and none was active against metallo-β-lactamase producers [59]. Swiss data on 150 carbapenemase-producing Enterobacterales, in which OXA-48-like and KPC-like enzymes each accounted for 32% and NDM-like for 24%, found overall susceptibility of 77% to meropenem-vaborbactam, 63% to ceftazidime-avibactam and 62% to imipenem-relebactam [60], appreciably lower than figures from KPC-dominated settings. Comparative in vitro testing further shows that activity differs by organism as well as enzyme: against carbapenem-resistant *P. aeruginosa*, imipenem-relebactam and ceftazidime-avibactam retained useful activity while most isolates were resistant to meropenem-vaborbactam [61]. Regional carbapenemase epidemiology therefore governs the utility of these agents directly, a consideration of particular weight in the Eastern Mediterranean, where OXA-48 and NDM predominate [8] rather than the KPC enzymes against which these combinations perform best.

A further finding is of direct relevance. In a collection of 284 carbapenem-resistant non-carbapenemase-producing Enterobacterales, the substantial subset described in Section 2.1, increased exposure of existing agents recovered a considerable proportion of susceptibility under EUCAST breakpoints: at standard dose, susceptibility to imipenem and meropenem was 45.1% and 14.8% respectively, rising to 68.3% and 67.7% at increased exposure [62]. For comparison, susceptibility in the same collection was 88.4% to ceftazidime/avibactam, 81.0% to imipenem/relebactam and 80.6% to meropenem/vaborbactam [62]; dose escalation of an existing carbapenem thus closed much, though not all, of the gap to agents costing substantially more. In the resistance phenotype driven by porin loss and efflux rather than enzymatic hydrolysis, dose optimisation recovers in vitro susceptibility categorisation that standard dosing forfeits; reclassification under an increased-exposure breakpoint indicates that a higher exposure is predicted to be effective, but is not itself direct evidence that clinical activity has been recovered, since clinical outcome data at the increased-exposure dose for this specific population are not reported in the same study. This demonstrates that the boundary between susceptible and resistant is partly a function of the dose assumed, and identifies the population in which the framework proposed here should be most consequential, while underlining that the newer β-lactam/β-lactamase inhibitor and colistin-based options discussed above are useful chiefly insofar as their organism- and enzyme-specific activity is confirmed locally (Section 4.5, preceding paragraph) rather than assumed from aggregate surveillance figures.

The dosing of newer agents carries its own PK/PD constraints and lies beyond the scope of this review, meriting separate evaluation.

### 4.6. Target Non-Attainment and the Emergence of Resistance

The preceding sections treat target attainment as a determinant of clinical cure. It also determines what happens to the pathogen population during treatment, and this second consequence bears directly on the argument of this review: if subtherapeutic exposure selects for elevated MICs, then dosing practice is among the causes of the resistance epidemiology described in Section 2, rather than simply a response to it.

Observational evidence consistent with this proposition has accumulated in recent years, though its design limits how strongly it can support a causal reading. A systematic review and meta-analysis of 21 observational studies (*n* = 4833; 2193 aggressive vs. 2640 conservative target), predominantly at moderate to serious risk of bias given their observational design, in which the aggressive target was defined as 100% fT > 4× MIC, found that attaining it was associated with a higher clinical cure rate (OR 1.69; 95% CI 1.15–2.49) and a substantially lower risk of β-lactam resistance development (OR 0.06; 95% CI 0.01–0.29) [29]. Because target attainment was not randomised in these studies, disease severity, source control, infecting organism, infection duration and confounding by indication (sicker patients, or those failing therapy, may be more likely to have dosing intensified or de-intensified) could each contribute to the observed association independently of any causal effect of exposure on resistance selection; the meta-analysis cannot exclude this. Failure to attain the aggressive target was in turn associated with a markedly increased risk of microbiological failure (OR 26.08; 95% CI 8.72–77.95) [29]. Male sex, body mass index > 30 kg/m^2^, augmented renal clearance and an MIC above the clinical breakpoint were independent predictors of failure to attain the target, whereas prolonged or continuous infusion was the only protective factor identified [29]. The appearance of augmented renal clearance and elevated MIC in that list connects this section directly to Section 2.2 and Section 3.3: the two conditions under which target attainment is most at risk are also the two this review identifies as under-recognised. In a retrospective cohort of 256 patients contributing 628 Gram-negative isolates treated with cefepime, meropenem or piperacillin-tazobactam, a mean daily fAUC/MIC ≥ 494 was associated with a lower likelihood of any increase in MIC (*p* = 0.002) and of a two-fold increase (*p* = 0.004); *P. aeruginosa* carried a markedly elevated risk of MIC increase (OR 6.41; 95% CI 3.34–12.28) [63]. Exposure-optimised dosing of ceftazidime/avibactam achieving concentrations ≥ 4× MIC has likewise been associated with an approximately three-fold reduction in resistance emergence [64]. Raising PK/PD targets to similarly limit resistance development to the newer β-lactam/β-lactamase inhibitor combinations has been proposed as a strategy but remains to be established [65].

The mechanistic literature aligns with these observations in a way specific to the phenotype described in Section 2.1. Non-carbapenemase-producing carbapenem-resistant Enterobacterales (non-CP CRE) acquire resistance through a combination of β-lactamase production (ESBL or AmpC) and mutation or loss of outer membrane porins, most importantly OmpK36 in *K. pneumoniae* [66]. In a series of 54 such isolates, porin loss was identified in 46.3% alongside non-carbapenemase β-lactamase genes [67], and molecular characterisation of ertapenem-non-susceptible non-carbapenemase-producing *K. pneumoniae* has confirmed the combined contribution of ESBL or AmpC production, porin loss at both genetic and protein levels, and efflux pump overexpression [68]. Ertapenem is the carbapenem most sensitive to permeability-related mechanisms and is correspondingly the first to lose activity as porin function declines [68].

That this phenotype can be selected during treatment rather than only acquired is documented directly. In a persistent *K. pneumoniae* ST395 bloodstream infection not amenable to source control, whole-genome sequencing of three consecutive blood isolates demonstrated in vivo selection of carbapenem resistance under therapy through OmpK36 deletion [66]. Non-CP CRE has more generally been proposed to emerge through de novo mutation within carbapenem-susceptible Enterobacterales under antimicrobial selective pressure [69]. A cohort of recurrent ESBL-producing Enterobacterales bacteraemia provides a detailed mechanistic account. Non-CP CRE emerged from an ESBL background through IS26-mediated amplification of *blaOXA-1* and *blaCTX-M-1* group genes coupled with porin inactivation, the two events being linked rather than independent: modular translocatable units carrying the β-lactamase genes mobilised from a chromosomal IS26-bound transposon and inserted within porin genes, simultaneously raising β-lactamase copy number and inactivating the porin [9]. Both the resistance phenotype and the amplification were reproduced experimentally by serial passage of a clinical ST131 *E. coli* isolate in the presence of ertapenem, and the resulting resistance remained stable in the absence of continued carbapenem exposure [9].

The relationship between exposure and resistance is therefore not uniform across mechanisms, and the experimental serial-passage and single-patient in vivo evolution evidence cited above demonstrates biological plausibility for exposure-driven selection at the level of an individual isolate; it does not, by itself, establish that institutional dosing practice causes population-level MIC drift, which is a distinct claim requiring longitudinal, institution-level data not yet available (Section 2.6). Acquisition of a carbapenemase is largely a matter of horizontal gene transfer and infection control. The non-carbapenemase pathway arises instead through incremental, selectable changes in the treated organism itself, and it is here that dosing decisions plausibly exert selective pressure. The sequence is also stepwise rather than binary, since porin loss and efflux upregulation raise the MIC by degrees and affect ertapenem before meropenem and imipenem.

This produces a feedback relationship rather than a linear one. Suboptimal exposure selects for incremental MIC elevation; elevated MIC raises the exposure required for target attainment; and if dosing practice does not adjust, the deficit widens. Whether this loop operates at the population level, and over what timescale, has not been demonstrated, and the proposition should be read as a hypothesis consistent with the available evidence rather than an established finding. It does, however, indicate that the carbapenem floor and the dosing practices described in this section may not be independent phenomena.

### 4.7. Comparative Summary of Strategies

The comparison exposes an inverse relationship: the strategies with the heaviest infrastructure requirements do not carry the strongest outcome evidence. The intervention with the weakest evidence base, MIC-guided empirical protocols, is also the one that has never been formally evaluated, so absence of evidence here reflects absence of trials rather than negative findings.

## 5. Integrating Surveillance with Clinical Practice

### 5.1. Translating Local MIC Data into Dosing Protocols

Antibiogram reports are conventionally presented as susceptibility percentages, answering the question “can this agent be used?” As shown in Section 3, however, the pertinent clinical question is frequently “at what dose should this agent be used?” To close this gap, institutional antibiograms should report MIC distributions alongside susceptibility rates, in particular MIC_50_ and MIC_90_ values.

The MIC90 is a useful screening parameter: a regimen can be tested for probability of target attainment (PTA) at the local MIC90 as a single, conservative reference point, giving a rapid first indication of whether a candidate regimen is likely to be adequate. This MIC90-based PTA check is distinct from, and does not substitute for, the cumulative fraction of response (CFR) evaluation described in Section 4.4, which requires the complete local MIC–frequency distribution rather than the MIC90 alone; an MIC90-adequate regimen can still leave a clinically relevant fraction of the distribution above target, particularly if that distribution is right-skewed. Used in this more limited, screening role, and followed by full-distribution CFR modelling before adoption, local MIC surveillance data can inform an institutional empirical protocol (Section 4.4). Surveillance data thereby cease to be a passive epidemiological report and become an active clinical decision instrument.

A parallel case can be made for reporting susceptibility at increased exposure alongside standard-dose categories where breakpoint systems permit it. As shown in Section 4.5, this distinction has practical consequence: among non-carbapenemase-producing carbapenem-resistant isolates, the proportion classified as susceptible more than quadruples for meropenem when increased exposure is assumed [62]. An antibiogram reporting only standard-dose susceptibility discards this information before it reaches the prescriber. A similar conclusion was reached in the vancomycin literature, where MIC creep was assessable only through local susceptibility profiles, with MIC-based antibiograms deemed essential to local management [14].

**Figure 3:**
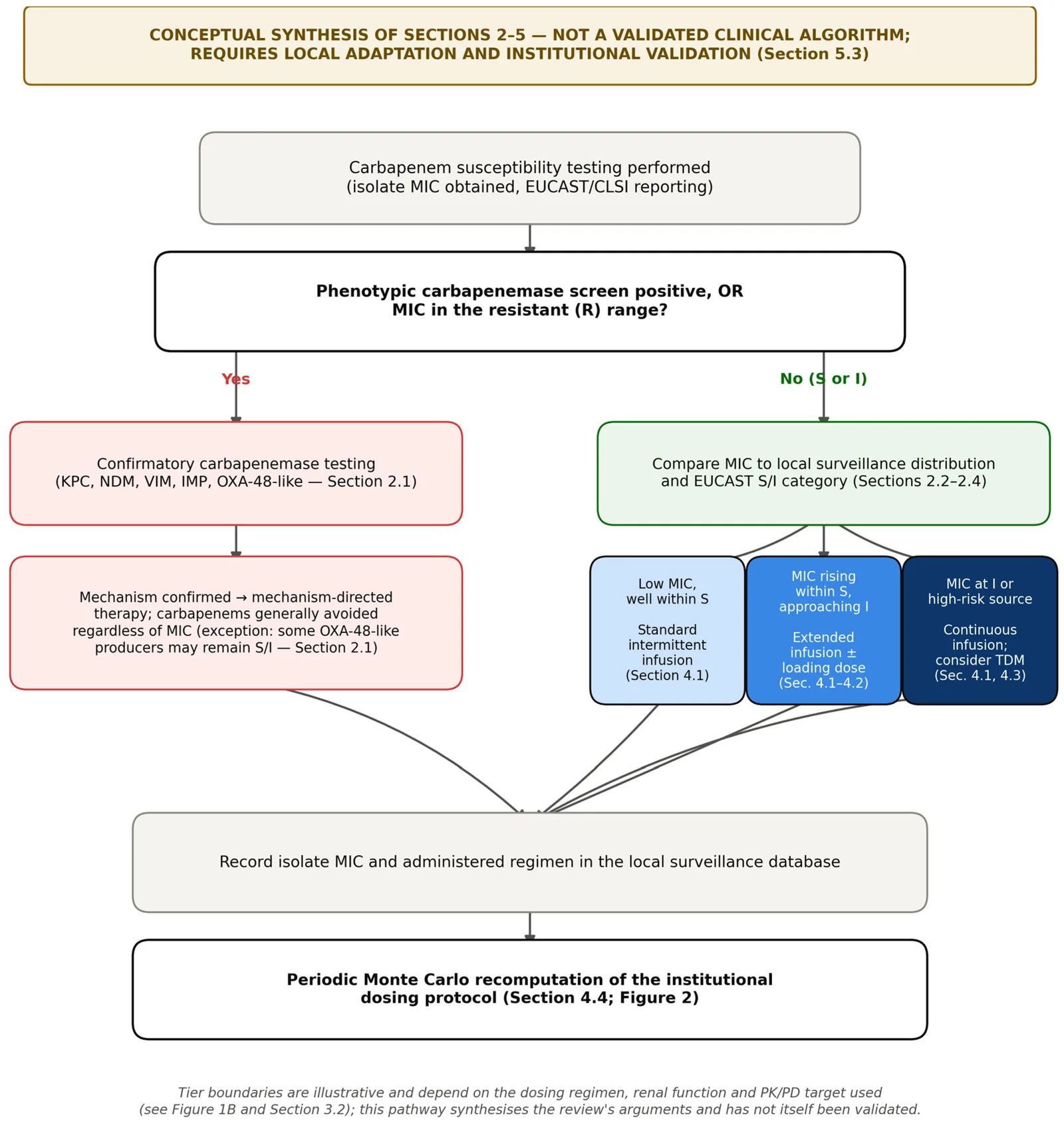
Conceptual decision-support framework integrating carbapenemase testing, MIC-tiered dose optimisation and local surveillance feedback (synthesised from Section 2, Section 3, Section 4 and Section 5 of this review; not a validated clinical algorithm). Box colour indicates the decision pathway: red = carbapenemase-positive, mechanism-directed pathway; green = MIC-based comparison step; light blue/mid blue/dark blue = increasing dosing-intensity tier (standard intermittent, extended, and continuous infusion, respectively); grey = data-recording and periodic-recomputation steps.

Figure 3 synthesises the concepts developed in Section 2, Section 3 and Section 4 into a single conceptual pathway, from carbapenemase testing through MIC-tiered dose optimisation to local surveillance feedback; it is offered as an integrative summary of the arguments made in this review, not as a validated clinical algorithm.

### 5.2. The Role of Antimicrobial Stewardship (AMS) Programmes

Dose optimisation is formally recognised within antimicrobial stewardship frameworks; the CDC core elements list dose optimisation explicitly among pharmacy-based interventions, alongside intravenous-to-oral conversion and duplicative therapy alerts, and include selective susceptibility reporting among microbiology-based interventions [70]. In practice, however, AMS activity has concentrated on agent selection and duration of therapy, with dosing receiving comparatively less attention. Yet inadequate dosing leads both to treatment failure and to selection of resistance during therapy, directly conflicting with the core objectives of AMS.

Dose optimisation is an attractive stewardship target on two counts. First, prolonged infusion of β-lactams is among the interventions amenable to pharmacist-led protocols operating under collaborative practice agreements, alongside renal dose adjustment and aminoglycoside or vancomycin monitoring; such pharmacy-led dosing strategies have been associated with higher rates of adequate serum concentrations, cost savings and improved treatment outcomes [71]. Second, institutional standardisation of extended infusion requires no new drug acquisition and can be implemented at essentially no marginal cost, a consideration of some importance where formulary budgets constrain access to newer agents.

Collaboration between the microbiology laboratory, the infectious diseases service and clinical pharmacy is necessary, since MIC-guided dosing requires the laboratory to report data it does not conventionally release, the pharmacy to act on it, and the clinical service to accept protocolised dosing. The infection control physician is positioned at this three-way interface and may serve a coordinating role.

AMS implementation is itself uneven. Baseline assessments in resource-constrained settings have documented inconsistent leadership commitment, limited resource allocation and fragmented education as recurring barriers [72]. Where AMS infrastructure is still being established, embedding dose optimisation from the outset may be more efficient than retrofitting it later.

### 5.3. Regional Context: The Eastern Mediterranean and Cyprus

The Eastern Mediterranean Region is characterised both by high carbapenem resistance rates and by limited surveillance capacity [3]. Carbapenem resistance determinants show heterogeneous distribution across countries in the region [8]. Regional trends have not improved: at the EU/EEA level, the population-weighted mean percentage of carbapenem resistance among invasive *K. pneumoniae* isolates has shown a significantly increasing trend, and the estimated incidence of carbapenem-resistant *K. pneumoniae* bloodstream infection reached 3.97 per 100,000 population in 2023, 57.5% higher than the 2019 baseline, with a country range of 0.00–21.44 [73]. Resistance percentages follow a north-to-south and west-to-east gradient, with the highest values reported by countries in southern and south-eastern Europe [73].

Cyprus requires a distinction that is easily missed. The EARS-Net figures cited earlier [2] derive from the Republic of Cyprus. Northern Cyprus, where this review was prepared, participates in neither EARS-Net nor the CAESAR network, and falls outside the WHO Eastern Mediterranean Region reporting framework as well [3]. It is therefore absent from all three of the surveillance systems that would ordinarily describe it, and its resistance epidemiology is represented in the literature only by individual institutional reports. The single published series from a university hospital in Northern Cyprus, covering isolates collected up to 2014, reported carbapenem resistance in *P. aeruginosa* and *K. pneumoniae* at lower rates than contemporaneous regional studies, and its authors called explicitly for multicentre work to establish resistance patterns and underlying mechanisms in Northern Cyprus [74]. That call has not been answered in the intervening decade.

This is not solely a local problem, and it is the reason the argument of Section 5.1 matters more here than elsewhere. Where a jurisdiction is absent from international surveillance, no external dataset can substitute for institutional MIC data: the antibiogram is not merely the most convenient source of information on which to base dosing, it is the only one. The corollary is that the framework proposed in this review cannot be evaluated in Northern Cyprus until MIC-resolved institutional series are generated and published, and that generating them is a prerequisite rather than an optional extension; this section is accordingly best read as identifying a research and surveillance agenda for the region rather than as a basis, on its own, for specific empirical treatment recommendations, which the absence of local data cannot supply regardless of the strength of the general PK/PD argument made elsewhere in this review. Comparable series are now appearing from tertiary centres in Türkiye [75], and analogous single-centre surveillance from regional hospitals in Greece has demonstrated what such reporting can establish about local resistance dynamics [76].

Where TDM infrastructure is not widely available, MIC-guided empirical protocols (Section 4.4) represent a more realistic priority than individual TDM. This should be regarded as a hypothesis about feasibility rather than an established finding; formal assessment of TDM availability across the region would be a valuable contribution in its own right.

## 6. Evidence Gaps and Future Directions

Several interrelated gaps emerge from the literature reviewed here.

MIC shift is not systematically monitored. Because major surveillance programmes rest on binary susceptible/resistant classification, MIC shifts occurring within the susceptible category remain invisible. Empirical testing of the carbapenem floor framework proposed here requires multicentre, longitudinal surveillance studies in which MIC distributions are reported as a continuous variable. The vancomycin experience is cautionary in this regard: pooled international analysis found no evidence of MIC creep [13], while local analyses suggested it is a regional phenomenon [14]. The same may prove true of carbapenems, which is itself an argument for local rather than aggregated surveillance.

PK/PD targets lack standardisation. A wide range of targets, from 40% fT > MIC to 100% fT > 4 × MIC, is in use, with no consensus on which target applies to which patient. This heterogeneity also weakens the comparability of TDM studies.

Evidence on hard clinical endpoints remains equivocal. Although TDM and prolonged infusion strategies consistently improve target attainment, their effects on mortality remain unproven: BLING III missed significance on its primary endpoint despite enrolling 7202 patients [40], MERCY found no benefit [37], DOLPHIN found no difference in its primary outcome [53], and the TDM meta-analysis showed no mortality effect [52,54].

A more productive question than trial size may be whether these interventions should be tested in selected populations, where the pharmacological rationale predicts the effect should concentrate. The DOLPHIN subgroup analysis is suggestive, identifying benefit in patients with SOFA below 8 [55]. No trial to date has stratified randomisation by pathogen MIC, the variable that on PK/PD grounds should most directly determine whether dose optimisation can matter. A trial enrolling only patients with pathogens at or above the local MIC90 would test the present hypothesis more efficiently than a further unselected trial of comparable size.

Jurisdictions outside international surveillance networks: Territories absent from EARS-Net, CAESAR and WHO regional reporting, of which Northern Cyprus is one (Section 5.3), have no external dataset against which to calibrate empirical dosing. For these settings the institutional antibiogram is the only available input, which makes MIC-resolved local reporting not an enhancement but a precondition for evidence-based dosing.

Applicability in resource-limited settings: The bulk of the current literature originates from high-income centres with TDM infrastructure. Studies evaluating the effectiveness of feasible, low-cost alternatives, protocols based on local MIC data, and institutional standardisation of extended infusion, in regions such as the Eastern Mediterranean are limited.

Institution-specific stability validation is not standard practice. As shown in Section 4.1, carbapenem stability varies by generic product, concentration and ambient temperature [43,44,45], yet extended-infusion protocols are frequently adopted from published guidance without local verification. This gap is likely to be most consequential in warm climates.

The dose-dependence of the susceptibility category is under-exploited. The demonstration that increased exposure raises imipenem susceptibility from 45.1% to 68.3% and meropenem from 14.8% to 67.7% among non-carbapenemase-producing carbapenem-resistant isolates [62] implies that a meaningful fraction of what is currently reported as resistance is conditional on the dose assumed. Whether reporting susceptibility at increased exposure alongside standard-dose categories would change prescribing, and outcomes, has not been evaluated prospectively.

The exposure–resistance loop is untested at population level. Individual-patient data associate aggressive target attainment with reduced resistance emergence [29,63], and the non-carbapenemase phenotype is known to be selectable under treatment [66]. Whether institution-wide adoption of prolonged infusion measurably slows upward drift of the local MIC distribution has not been examined. Such a study is feasible with existing surveillance infrastructure, requiring only that MIC be recorded as a continuous variable before and after a dosing-protocol change.

A research gap at the surveillance–dosing interface: Whether empirical dosing protocols adapted to local MIC distribution improve clinical outcomes has not been tested directly. This is both feasible and, on the evidence assembled here, a priority.

## 7. Conclusions

The global and regional rise in carbapenem resistance raises a second question alongside that of which agent to use: what exposure to achieve. MIC shifts occurring within the susceptible category, the phenomenon proposed here as the carbapenem floor, may erode the capacity of standard regimens to deliver target exposure without appearing in surveillance reports. Pharmacokinetic variability in the critically ill compounds this.

Extended and continuous infusion, loading-dose strategies, therapeutic drug monitoring and empirical protocols based on local MIC data are complementary rather than alternative approaches, and their effectiveness depends on connecting resistance surveillance to dosing decisions. The relationship runs in both directions: resistance epidemiology determines what exposure is required, while exposure achieved during treatment appears in turn to influence resistance selection (Section 4.6). Extending antibiogram reports to include MIC distributions, and channelling those data into dosing protocols through antimicrobial stewardship programmes, requires no new drug acquisition or TDM assay infrastructure and is particularly relevant where TDM infrastructure is limited—though, as Section 4.4 details, it still requires real investment in laboratory capacity, data management and pharmacy time.

Three tiers of confidence should be kept distinct in reading these conclusions. Extended and continuous infusion following an adequate loading dose, and the general principle that dosing should account for local MIC distributions and renal function, rest on the strongest evidence base and align with current guideline recommendations (Section 4.1). Structured therapeutic drug monitoring and MIC-guided empirical protocols are conditional strategies, plausible and increasingly recommended in selected patients, but not yet supported by consistent hard-outcome evidence (Section 4.3 and Section 4.4). The carbapenem floor itself, the proposed exposure–resistance feedback loop (Section 4.6), and the applicability of this framework to settings outside international surveillance networks (Section 5.3) remain untested research hypotheses, offered here to motivate the longitudinal, MIC-resolved surveillance without which none of them can be confirmed or refuted.

Prolonging the clinical lifespan of the carbapenems depends not only on developing new agents, but on administering existing ones at adequate exposure.

**Key Points**

- Carbapenem MICs can drift upward within the “susceptible” range without triggering any breakpoint-based alarm—proposed here as the “carbapenem floor,” a hypothesis-generating framework, not yet an empirically established trend (Section 2.2).
- Because carbapenem PK/PD targets scale directly with MIC, this invisible drift can erode target attainment before an isolate is ever reported as resistant; augmented renal clearance in critically ill patients compounds the problem (Section 3).
- This framework applies only to non-enzymatic resistance (porin loss, efflux, β-lactamase amplification). When a carbapenemase (KPC, NDM, VIM, IMP, OXA-48-like) is present, mechanism-specific testing and mechanism-directed therapy—not dose optimisation—should guide treatment (Section 2.1).
- The inoculum effect is a separate, testing-condition-dependent mechanism that can also elevate the effective MIC at high bacterial burden, independent of any true population-level MIC shift (Section 2.5).
- Dose optimisation (extended/continuous infusion, loading doses, therapeutic drug monitoring) and EUCAST’s ongoing consultation on lowering carbapenem breakpoints are complementary, not competing, responses to this problem (Section 2.4).
- No single strategy reviewed here is supported strongly enough to serve as a universal default; each carries specific evidence limitations, infrastructure requirements and applicability constraints, summarised for comparison in the tables throughout this review.

## Figures and Tables

**Table 1:** Terminology: the carbapenem floor compared with related concepts in resistance surveillance.

Term	Definition	Relationship to the Carbapenem Floor
MIC creep	A retrospectively documented upward shift in a population’s MIC distribution over time (e.g., the vancomycin/S. aureus literature, Section 2.2).	The carbapenem floor is a prospective hypothesis about where such creep, if it occurs, would matter clinically—within the susceptible category, not at its boundary.
ECOFF (epidemiological cut-off value)	Separates wild-type isolates (no acquired or mutational resistance mechanism) from non-wild-type isolates; typically well below clinical breakpoints and unaffected by dosing considerations.	The floor concerns isolates that are already non-wild-type (Section 2.1) and asks whether their clinical breakpoint category still predicts achievable exposure.
EUCAST “susceptible, increased exposure” (I)	A fixed, dose-linked breakpoint zone (e.g., meropenem > 2–8 mg/L) requiring a higher-exposure regimen (Section 2.4).	A static breakpoint category, whereas the floor describes a dynamic tendency for isolates to accumulate within or below it over time.
Carbapenemase-mediated resistance	Acquisition of an enzyme (KPC, NDM, VIM, IMP, OXA-48-like) that inactivates carbapenems (Section 2.1).	A largely distinct, more urgent problem: mechanism-specific testing and therapy—not dose optimisation—apply once a carbapenemase is confirmed. The floor concept applies to non-enzymatic resistance.
Inoculum effect	A testing-condition-dependent rise in effective MIC at high bacterial burden or inoculum density (Section 2.5).	A separate mechanism that can mimic or compound an apparent floor-related MIC shift without reflecting a true population-level drift.

**Table 2:** PK/PD targets for β-lactam classes and their implications for dosing.

β-Lactam Class	Target fT > MIC (Non-Neutropenic)	Post-Antibiotic Effect vs. Gram-Negatives	Practical Implication
Penicillins	50–60% [27]	Absent [28]	Frequent dosing or extended infusion
Cephalosporins	60–70% [27]	Absent [28]	Extended infusion favourable
Carbapenems	40–50% [27]; 100% in select critically ill populations, e.g., ARC [5]; 100% fT > 4× MIC proposed for resistance suppression on observational evidence [29]	Present [28]	Lowest baseline threshold of the three classes; higher targets are population- and evidence-tier specific, not universal (Section 3.1)

**Table 3:** Reported stability of carbapenem solutions under varying conditions.

Agent	Concentration	Temperature	Stability Duration	Reference
Meropenem (generic, Hospira)	5 mg/mL	25–35 °C	up to 8 h	[44]
Meropenem (generic, Hospira)	5 mg/mL	40 °C	5 h	[44]
Meropenem (generic, brand A)	10 mg/mL	25 °C	up to 10 h	[45]
Meropenem (generic, brand A)	10 mg/mL	30 °C	5 h	[45]
Meropenem (generic, brand A)	10 mg/mL	35 °C	4.5 h	[45]
Meropenem (generic, brand A)	20 mg/mL	25 °C	6 h	[45]
Meropenem (generic, brand A)	20 mg/mL	30 °C	5 h	[45]
Meropenem (generic, brand A)	20 mg/mL	35 °C	3 h (10% loss by 1 h)	[45]
Meropenem (generic, brand B)	10 mg/mL	25 °C	9 h	[45]
Meropenem (generic, brand B)	10 mg/mL	30 °C	5 h	[45]
Meropenem (generic, brand B)	10 mg/mL	35 °C	3 h	[45]
Meropenem	1% (cooled elastomeric device)	cooled	90.3% recovery at 24 h	[47]
Imipenem	5 mg/mL	25, 30, 40 °C	6 h	[46]
Imipenem	10 mg/mL	25 °C	3–6 h (brand-dependent)	[46]
Imipenem	10 mg/mL	30, 40 °C	<1 h	[46]
Carbapenems (concentrated)	6.4 g/100 mL	25 °C	>10% degradation in ≤5–6 h	[43]

Note: values are drawn from independent studies using different generic products, concentrations and assay conditions, and are therefore not directly comparable with one another; the heterogeneity is itself the point (see text). Table S1 (Supplementary Materials) gives, for each cited study, the diluent, container/administration device, analytical method and stability criterion where these could be confirmed from the accessible source; these parameters should be checked against the primary source before a given stability window is adopted locally, consistent with the institution-specific validation recommended in the text.

**Table 4:** Dose optimisation strategies: evidence, requirements and applicability.

Strategy	Effect on Target Attainment	Evidence on Clinical Outcomes	Additional Infrastructure Required	Best-Suited Patients
Extended infusion (3–4 h)	Improves PTA, particularly at higher MIC [5]	SSC 2026: strong recommendation, moderate certainty [36]; reduces resistance emergence [29]	Infusion pumps; local stability validation	Broad ICU use; high-MIC pathogens
Continuous infusion	Greatest fT > MIC gain [5]	BLING III: OR 0.91, *p* = 0.08 (adjusted *p* = 0.04); improved cure [40]. MERCY: no benefit [37]	As above, plus cooling or frequent bag renewal [44,47]	Sepsis in ICU; agent-dependent (meropenem feasible, imipenem less so)
Loading dose	Shortens time to target with continuous infusion	Extrapolated from PK principles [51]	None	All patients starting continuous infusion
TDM/MIPD	Consistently improves attainment (RR 1.85) [52]	No mortality benefit overall [52,53,54]; subgroup signal at SOFA < 8 [55]. SSC 2026: conditional suggestion, very low certainty [36]	Assay capability; turnaround time; dosing software	ARC, CRRT, high-MIC pathogens, non-response—an evidence-informed prioritisation, not a validated algorithm (Section 4.3)
MIC-guided empirical protocol	Population-level CFR optimisation [4]	Not tested prospectively	MIC-resolved antibiogram; popPK model selection; simulation expertise	Institution-wide, where TDM unavailable

## Data Availability

No new data were created or analyzed in this study. Data sharing is not applicable to this article.

## Supplementary Materials

The following supporting information can be downloaded at: https://www.mdpi.com/article/10.3390/antibiotics15090885/s1, Table S1: Diluent, container/administration device, analytical method and stability criterion for the carbapenem stability studies summarised in Table 3 of the main text; Table S2: Structured evidence table for the randomised trials, meta-analyses and simulation studies underlying the review’s central clinical claims.
