# Supplementary material for: Rising Carbapenem MICs and Clinical Dose Optimization: Bridging Resistance Surveillance and Pharmacokinetic/Pharmacodynamic Strategies
Source: Antibiotics (Basel). 2026 Sep 9;15(9):885. doi: 10.3390/antibiotics15090885 (PMC13603404; doi:10.3390/antibiotics15090885)
Supplement: Supplementary file 1 [file antibiotics-15-00885-s001.zip › antibiotics-4536325-supplementary.pdf]

Supplementary Materials

Manuscript: "Rising Carbapenem MICs and Clinical Dose Optimization: Bridging Resistance Surveillance and Pharmacokinetic/Pharmacodynamic Strategies"

Table S1. Diluent, container/administration device, analytical method and stability criterion for the carbapenem stability studies summarised in Table 3 of the main text.

Reference	Diluent	Container / Administration Device	Analytical Method	Stability Criterion
[43] Berthoin et al., 2010	Not confirmed*	Not confirmed*	Not confirmed*	Not confirmed*
[44] Franceschi et al., 2014	Not confirmed*	Not confirmed*	Not confirmed*	Not confirmed*
[45] Katip et al., 2015	Not confirmed*	Not confirmed*	Not confirmed*	Not confirmed*
[46] Sornsuvit et al., 2021	0.9% sodium chloride (normal saline)	PVC infusion bags	HPLC, reverse-phase (C18 column), isocratic elution 1.0 mL/min, UV detection at 300 nm	≥90% of initial (0 h) concentration remaining (USP criterion)
[47] Manning et al., 2014	0.9% sodium chloride (normal saline)	Elastomeric infusion device (Baxter LV10, 240 mL capacity)	LC-MS, ertapenem sodium used as internal standard	>90% recovery (pharmacopeial standard); delivery of approximately 95% of the maximum deliverable dose over 24 h was additionally assessed as the clinically relevant threshold

** Diluent, container/administration device, analytical method and stability criterion for references [43], [44] and [45] could not be independently confirmed from the accessible portions of the primary source during this revision (full-text access was not available). Readers should consult these primary sources directly before adopting a specific stability window in local practice. All five studies used HPLC-based or LC-MS-based quantification methods typical of this literature; only the two studies above could be confirmed with full methodological specificity within the time available for this revision.*

Table S2. Structured evidence table for the randomised trials, meta-analyses and simulation studies underlying the review's central clinical claims.

Study	Design	Population	Principal Endpoint / Finding	Key Limitation
Wise et al., 2024 [1]	Global surveillance programme (ATLAS), 2018–2022	Clinical isolates of WHO priority bacterial pathogens, multiple countries	Rising carbapenem- and difficult-to-treat resistance across the surveillance period	Passive, industry-affiliated surveillance network; participating-laboratory selection bias
Diaz et al., 2018 [13]	Systematic review and meta-analysis	MRSA isolates (vancomycin MIC creep literature)	Evidence of upward vancomycin MIC drift over time within the susceptible range	Different drug class (glycopeptide); used here as a methodological analogy, not direct carbapenem evidence
Udy et al., 2014 [34]	Multicentre observational study	Critically ill ICU patients with normal plasma creatinine concentration	High prevalence of augmented renal clearance in this population	Observational, cross-sectional prevalence estimate; no interventional or outcome data
Luo et al., 2025 [5]	Monte Carlo PK/PD simulation (population PK model of Gijsen et al., 2022)	Simulated critically ill patients with augmented renal clearance (CrCl 140–200 mL/min)	Required infusion duration for 100% fT>MIC rises with MIC; standard intermittent infusion becomes inadequate above a threshold MIC	Simulation-based (not a clinical trial); single PK model and target; no loading dose modelled
Dulhunty et al., 2013 (BLING I) [38]	Multicentre, double-blind RCT	Severe sepsis, ICU	Continuous infusion beta-lactams improved a secondary clinical-cure endpoint	Small sample; underpowered for mortality; secondary-endpoint finding
Dulhunty et al., 2015 (BLING II) [39]	Multicentre RCT	Severe sepsis, ICU	No significant difference between continuous and intermittent infusion for ICU-free days	Composite/surrogate endpoint; no mortality benefit demonstrated
Monti et al., 2023 (MERCY) [37]	Multicentre RCT	Critically ill patients with sepsis	No significant difference in the primary composite endpoint between continuous and intermittent meropenem	Composite endpoint; trial underpowered to detect a mortality difference alone
Dulhunty et al., 2024 (BLING III) [40]	Large multicentre RCT	Critically ill patients with sepsis, multiple countries	No significant difference in 90-day mortality (primary outcome); signals in some secondary analyses	Non-significant primary outcome; average trial effect across an unselected population, not a targeted subgroup
Ewoldt et al., 2022 (DOLPHIN) [53]	Multicentre RCT	Critically ill patients receiving beta-lactams or ciprofloxacin	No significant difference in primary clinical outcome between TDM-guided and standard dosing	Neutral primary result; population-level effect may mask benefit in specific subgroups
Ewoldt et al., 2023 (DOLPHIN subgroup) [55]	Secondary/exploratory subgroup analysis of the DOLPHIN RCT	Subgroup stratified by baseline SOFA score	Suggestion of benefit from model-informed precision dosing in patients with SOFA <8	Exploratory and hypothesis-generating; not powered for subgroup inference; should not guide patient selection alone
Hagel et al., 2022 [56]	RCT	Patients with sepsis receiving piperacillin/tazobactam	TDM-based dose optimization reduced sepsis-related organ dysfunction	Single-agent (piperacillin/tazobactam) findings extrapolated to carbapenem TDM by analogy, not direct evidence
Pai Mangalore et al., 2022 [52]	Systematic review and meta-analysis	Critically ill patients receiving beta-lactam TDM	TDM associated with improved outcomes in some pooled analyses	Substantial heterogeneity; predominantly observational constituent studies

Gatti et al., 2024 [64]	Systematic review and meta-analysis	Critically ill patients with Gram-negative infections receiving beta-lactams	Aggressive PK/PD target attainment associated with improved clinical efficacy	Predominantly observational constituent studies; risk of confounding by indication
Prescott et al., 2026 (Surviving Sepsis Campaign) [36]	International clinical practice guideline	Adults with sepsis or septic shock	Recommends optimized beta-lactam dosing (extended/continuous infusion after a loading dose) as part of management	Guideline synthesis; certainty of evidence varies by individual recommendation and is not detailed here

Studies are ordered as first cited in the main text. This table summarises, rather than replaces, the fuller discussion of evidence quality and limitations given for each study in the relevant section of the main text.
